# In reply to Letter to the Editor from Paudel: Comment on “Changes in iPSC-Astrocyte Morphology Reflect Alzheimer’s Disease Patient Clinical Markers”

**DOI:** 10.1093/stmcls/sxaf031

**Published:** 2025-05-15

**Authors:** Noel J Buckley, Helen A Rowland

**Affiliations:** Department of Psychiatry, University of Oxford, Oxford OX3 7JX, United Kingdom; Kavli Institute for Nanoscience Discovery, University of Oxford, Oxford OX1 3QU, United Kingdom; Department of Psychiatry, University of Oxford, Oxford OX3 7JX, United Kingdom; Kavli Institute for Nanoscience Discovery, University of Oxford, Oxford OX1 3QU, United Kingdom

Paudel comments on our recent publication, “*Changes in iPSC-Astrocyte Morphology Reflect Alzheimer’s Disease Patient Clinical Markers*,”^1^ and highlights several aspects of our study that represent a major advancement in Alzheimer’s disease (AD) research. We are grateful for Paudel’s comments and recognition of the importance of our study. Here we build on Paudel’s commentary.

It is widely recognized that induced pluripotent stem cells (iPSC)-derived cells can model general aspects of AD pathogenesis, and many patient versus control studies have shown AD patient-specific differences in cellular and molecular phenotype. To date, most studies have understandably focused on neuronal phenotypes. The large number of studies in neurons indicates numerous phenotypes, including elevated levels and ratios of Aβ42, Aβ40, and Aβ38.^2-4^ Other phenotypes include increases in total and phosphorylated tau,^5^ GSK3β,^6^ and synaptic,^7^ oxidative stress, and mitochondrial changes.^8,9^ These studies include iPSC neurons derived from familial AD patients bearing pathogenic mutations in APP, PSEN1, PSEN2, but have been demonstrated in sporadic AD patients, with notably more variation in phenotype,^3^ reflecting diverse etiology leading to disease pathogenesis.^10^

In recent years, more research has been published on astrocytic phenotypes deriving astrocytes from familial and sporadic lines, notably focusing on AD risk genes such as APOE. These studies have also demonstrated wide-ranging phenotypes associated with AD, including key processes such as neurotrophic support,^11^ morphology changes,^12^ Aβ production and clearance, inflammatory response,^13,14^ cholesterol and lipid metabolism,^7,15^ oxidative stress, and calcium regulation.^16,17^ These studies uncover cell-type specific insight into the mechanisms behind AD pathology, but cannot address the complexity and diversity of disease phenotypes across patients, with most of these studies typically using anywhere from 1 to 4 patient lines versus control comparison. However, these studies do not attempt to correlate the cellular and molecular phenotype with the clinical presentation of the patients from whom the iPSCs were derived.

Perhaps, the most significant finding of our study is the demonstration that iPSC-derived astrocytes derived from a panel of AD patients not only provide a general cellular model of AD but also exhibit phenotypes that correlate with patient clinical data. Specifically, we show that astrocytes from AD patients with high and low levels of the AD biomarker, CSF YKL-40, can be stratified based on how they undergo structural changes in response to Aβ. This builds upon an earlier study where we showed cognitive vulnerability correlated with individual cellular vulnerability to extrinsic amyloid-β in vitro, as measured by synapse loss and function from the same patient cohorts.^18^ Correlations between iPSC-neuron and CSF Aβ were also observed, where both of these findings have been echoed in a similar study revealing significant associations between specific Aβ and tau species in iPSC neurons and the levels of Aβ plaque and neurofibrillary tangle deposition in the brain and the trajectory of cognitive decline.^19^

These studies are important for 2 principle reasons. First, they allow patient stratification by providing cellular models of disease that reflect the heterogeneity of complex disorders, which can, in turn, lead to the discovery of early biomarkers for disease progression. Second, identification of patient-specific cellular phenotypes can lead to the development of novel therapeutics that target specific subsets of patients, potentially leading to tailored treatment strategies based on cellular phenotypes.

Moreover, in our study, we add another dimension to this by demonstrating the utility of AI to correlate astrocyte morphology with patient clinical data. AI is increasingly being deployed to interrogate cellular, molecular, and clinical data sets, aiding earlier detection and diagnosis (such as exampled by our study, brain imaging,^20^ and biomarker discovery^21^), understanding of the underlying disease mechanisms, and drug discovery. Integration of multi-modal data by AI offers the ability to gain a comprehensive understanding of disease complexity. Moreover, AI has been used to impute gene expression profiles from cellular morphology in iPSC models of amyotrophic lateral sclerosis and expedite correlations between cellular and patient phenotype.^22^ This potential of AI to predict changes in molecular phenotype from cellular phenotype is an exciting prospect of these approaches, with huge implications for novel drug screening platforms.

In order to fully harness the potential of iPSCs to model disease heterogeneity, several challenges remain to be overcome. Scale is important; robust correlations require a large sample size to distinguish signal from noise to uncover subtle phenotypes. Large patient cohorts with attendant longitudinal data, coupled with an ability to measure cellular phenotype in multiple iPSC lines, are required. AI is revolutionizing the analysis of cellular phenotype and will change the face of drug discovery, and as shown by our study, provides a valuable tool in stratifying disease phenotype. We anticipate a surge of reports that use and extend these approaches to use iPSCs to interrogate the relationship between cellular and patient phenotype in multiple diseases, which, in turn, will greatly enhance patient stratification and personalized medicine.

## References

[1] Rowland HA , Miller G, Liu Q, et al Changes in iPSC-astrocyte morphology reflect Alzheimer’s disease patient clinical markers. Stem Cells. 2024;43(3):sxae085. https://doi.org/10.1093/stmcls/sxae085 PMC11907432 39704342

[2] Muratore CR , Rice HC, Srikanth P, et al The familial Alzheimer’s disease APPV717I mutation alters APP processing and Tau expression in iPSC-derived neurons. Hum Mol Genet. 2014;23:3523-3536. https://doi.org/10.1093/hmg/ddu064 24524897 PMC4049307

[3] Israel MA , Yuan SH, Bardy C, et al Probing sporadic and familial Alzheimer’s disease using induced pluripotent stem cells. Nature. 2012;482:216-220. https://doi.org/10.1038/nature10821 22278060 PMC3338985

[4] Sproul AA , Jacob S, Pre D, et al Characterization and molecular profiling of PSEN1 familial Alzheimer’s disease iPSC-derived neural progenitors. PLoS One. 2014;9:e84547. https://doi.org/10.1371/journal.pone.0084547 24416243 PMC3885572

[5] Ochalek A , Mihalik B, Avci HX, et al Neurons derived from sporadic Alzheimer’s disease iPSCs reveal elevated TAU hyperphosphorylation, increased amyloid levels, and GSK3B activation. Alzheimers Res Ther. 2017;9:90. https://doi.org/10.1186/s13195-017-0317-z 29191219 PMC5709977

[6] Hossini AM , Megges M, Prigione A, et al Induced pluripotent stem cell-derived neuronal cells from a sporadic Alzheimer’s disease donor as a model for investigating AD-associated gene regulatory networks. BMC Genomics. 2015;16:84. https://doi.org/10.1186/s12864-015-1262-5 25765079 PMC4344782

[7] Lin YT , Seo J, Gao F, et al APOE4 causes widespread molecular and cellular alterations associated with Alzheimer’s disease phenotypes in human iPSC-derived brain cell types. Neuron. 2018;98:1141-1154.e7. https://doi.org/10.1016/j.neuron.2018.05.008 29861287 PMC6023751

[8] Kondo T , Asai M, Tsukita K, et al Modeling Alzheimer’s disease with iPSCs reveals stress phenotypes associated with intracellular Abeta and differential drug responsiveness. Cell Stem Cell. 2013;12:487-496. https://doi.org/10.1016/j.stem.2013.01.009 23434393

[9] Birnbaum JH , Wanner D, Gietl AF, et al Oxidative stress and altered mitochondrial protein expression in the absence of amyloid-beta and tau pathology in iPSC-derived neurons from sporadic Alzheimer’s disease patients. Stem Cell Res. 2018;27:121-130. https://doi.org/10.1016/j.scr.2018.01.019 29414602

[10] Rowland HA , Hooper NM, Kellett KAB. Modelling sporadic Alzheimer’s disease using induced pluripotent stem cells. Neurochem Res. 2018;43:2179-2198. https://doi.org/10.1007/s11064-018-2663-z 30387070 PMC6267251

[11] Watanabe H , Murakami R, Tsumagari K, et al Astrocytic APOE4 genotype-mediated negative impacts on synaptic architecture in human pluripotent stem cell model. Stem Cell Rep. 2023;18:1854-1869. https://doi.org/10.1016/j.stemcr.2023.08.002 PMC10545487 37657448

[12] Jones VC , Atkinson-Dell R, Verkhratsky A, Mohamet L. Aberrant iPSC-derived human astrocytes in Alzheimer’s disease. Cell Death Dis. 2017;8:e2696. https://doi.org/10.1038/cddis.2017.89 28333144 PMC5386580

[13] Gomes C , Huang KC, Harkin J, et al Induction of astrocyte reactivity promotes neurodegeneration in human pluripotent stem cell models. Stem Cell Rep. 2024;19:1122-1136. https://doi.org/10.1016/j.stemcr.2024.07.002 PMC11368677 39094561

[14] Sullivan MA , Lane SD, McKenzie ADJ, et al iPSC-derived PSEN2 (N141I) astrocytes and microglia exhibit a primed inflammatory phenotype. J Neuroinflammation. 2024;21:7. https://doi.org/10.1186/s12974-023-02951-2 38178159 PMC10765839

[15] Tcw J , Qian L, Pipalia NH, et al Cholesterol and matrisome pathways dysregulated in astrocytes and microglia. Cell. 2022;185:2213-2233.e25. https://doi.org/10.1016/j.cell.2022.05.017 35750033 PMC9340815

[16] Oksanen M , Petersen AJ, Naumenko N, et al PSEN1 mutant iPSC-derived model reveals severe astrocyte pathology in Alzheimer’s disease. Stem Cell Rep. 2017;9:1885-1897. https://doi.org/10.1016/j.stemcr.2017.10.016 PMC5785689 29153989

[17] Brezovakova V , Sykova E, Jadhav S. Astrocytes derived from familial and sporadic Alzheimer’s disease iPSCs show altered calcium signaling and respond differently to misfolded protein tau. Cells. 2022;11:1429. https://doi.org/10.3390/cells11091429 35563735 PMC9101114

[18] Ng B , Rowland HA, Wei T, et al Neurons derived from individual early Alzheimer’s disease patients reflect their clinical vulnerability. Brain Commun. 2022;4:fcac267. https://doi.org/10.1093/braincomms/fcac267 36349119 PMC9636855

[19] Lagomarsino VN , Pearse RV 2nd, Liu L, et al Stem cell-derived neurons reflect features of protein networks, neuropathology, and cognitive outcome of their aged human donors. Neuron. 2021;109:3402-3420.e9. https://doi.org/10.1016/j.neuron.2021.08.003 34473944 PMC8571042

[20] Yao Z , Wang H, Yan W, et al Artificial intelligence-based diagnosis of Alzheimer’s disease with brain MRI images. Eur J Radiol. 2023;165:110934. https://doi.org/10.1016/j.ejrad.2023.110934 37354773

[21] Winchester LM , Harshfield EL, Shi L, et al Artificial intelligence for biomarker discovery in Alzheimer’s disease and dementia. Alzheimers Dement. 2023;19:5860-5871. https://doi.org/10.1002/alz.13390 37654029 PMC10840606

[22] Atmaramani R , Dreossi T, Ford K, et al Deep learning analysis on images of iPSC-derived motor neurons carrying fALS-genetics reveals disease-relevant phenotypes. bioRxiv. https://doi.org/10.1101/2024.01.04.574270

